# The Dental Protective Association

**Published:** 1899-10

**Authors:** 


### Editorial.

### THE DENTAL PROTECTIVE ASSOCIATION.

THE recent decision of Judge Townsend, in the United States Circuit Court of New York, in favor of the International Tooth-Crown Company, has elicited wide interest in the dental profession, perhaps more than it intrinsically deserves, but it has awakened dentists from a lethargy that has held them too long; and if it has no other value, it will show, as never before, the folly of neglecting an organization that has for a series of years protected them without regard to membership.

The years that have elapsed since the contest over the use of vulcanized rubber in plates has caused dentists to forget that experience, and they have apparently settled down to the belief that never again would there be a repetition of that disagreeable period. That they have been rudely awakened by the shock of this recent decision is not to be regretted.

From the reports that come to us, it is evident that the New York dentists are being pressed severely, and are being forced to pay the International Tooth-Crown Company large sums of money. If the dentists of that State had given but a small proportion of these sums to the Dental Protective Association, they would not only have been richer in their bank accounts, but would have in part enabled that organization to do more effective work. What is true of New York will be equally true of every State, and the dentists of the country may as well make up their minds to adopt one of two courses,—either to pay for all crowns inserted during the life of the patents held by the company, also pay for a license, and, in addition, become the willing slave of a corporation, or they must become part of the Dental Protective Association. There can be no half-way course.

There has been much said and written prejudicial to the Protective Association by those not connected with it and, apparently, as an excuse for not becoming part of it. They will now have the opportunity of transferring their prejudice to a party whose interests lie diametrically in opposition to their own and those of the dental profession.

It is one of the anomalies of the human mind that it so frequently turns and rends those who seek to help. The history of humanity is full of this kind of ingratitude, and will continue to be full of it as long as the race exists on the earth. While this is true, it is none the less discouraging to find this feeling active in professional circles. The individual who sacrifices himself that the profession may advance through his efforts, becomes the target for abuse. The dental colleges that have made the dental profession have had to undergo a continual storm of criticism, degenerating oftentimes into positive abuse and slander. The Dental Protective Association organized to act as a shield against the encroachments of monopoly, has been met first by an apathetic indifference, and finally ending in false representations of the one man who has borne the burden of this organization from the beginning. Charges have been made of misappropriation of funds; of using the Dental Protective Association to develop a commercial enterprise in which he was interested. That these charges have had no foundation in fact, the reports of the Auditing Committees of both the American and National organizations fully testify; yet, in the face of these official statements, these charges have been repeated and re-repeated until many worthy men have felt that to rely on the Dental Protective Association was a dependence upon a support that would fail them in the hour of trial. The fact, ever present, that dentistry had been protected through a long series of years counted for nothing, and rather than aid in this protection they would take their chances outside of the organization, selfishly trusting that they would be protected by decisions of the courts procured by the Protective Association through a great expenditure of time, labor, and money. The time has now arrived when this protection will be withdrawn from this class, and all those outside of the Dental Protective Association will be forced to bow to the demands of the International Tooth-Crown Company. The chairman of the Dental Protective Association says in a recent editorial: "We speak advisedly when we state that every practitioner who unites with this organization will be taken care of and be protected against any claims for royalty." This entirely shuts out, and very properly, all those who are not in membership or do not immediately propose to avail themselves of the protection afforded by this organization.

The attitude of dentistry towards the Dental Protective Association has not been one to be commended; and by the course pursued much has been lost, not only in protection, but in that broader relation of interest,—true professional unity.

While it may be the eleventh hour, it is not too late to form a united front to oppose the arch-enemy of our household. If this be done promptly, there will soon be an end to the demand of these patent speculators.

Had this united front been in existence during the rubber controversy, Bacon could never have accomplished the results he achieved. Let those who do not wish to be placed in the same humiliating position of the dentists of that period, at once take measures to have their names enrolled, and by thus doing they will have accomplished a twofold object,—protection for themselves and will have given an added dignity to their profession.

